# Primary Intraosseous Cavernous Hemangioma of the Petrous Bone Involving the Jugular Fossa Mimicking a Jugular Paraganglioma: A Radiologic-Pathologic Correlation and Review of Relevant Literature

**DOI:** 10.7759/cureus.115906

**Published:** 2026-09-07

**Authors:** Ketki Kedar, Deepshikha Arora, Akshay K Rao, Avinash Dhok, Saikat Mitra

**Affiliations:** 1 Radiodiagnosis, All India Institute of Medical Sciences (AIIMS), Nagpur, IND; 2 Radiodiagnosis and Interventional Radiology, All India Institute of Medical Sciences (AIIMS), Nagpur, IND; 3 Pathology, All India Institute of Medical Sciences (AIIMS), Nagpur, IND

**Keywords:** computed tomography angiography, differential diagnosis, jugular foramen, paraganglioma, primary intraosseous cavernous hemangioma, skull base, temporal bone

## Abstract

Primary intraosseous cavernous hemangioma (PICH) is a rare benign vascular tumor that predominantly involves the vertebral column and calvarium, while skull base involvement, particularly the jugular fossa, is exceptionally uncommon. We report a histopathologically confirmed PICH centered in the petrous temporal bone showing jugular fossa and jugular foraminal involvement, radiologically mimicking a jugular paraganglioma. A 46-year-old man presented with progressive unilateral sensorineural hearing loss. Magnetic resonance imaging (MRI) demonstrated a heterogeneously enhancing jugular foramen lesion with cerebellopontine angle extension, whereas high-resolution computed tomography (HRCT) revealed characteristic coarse trabecular ("honeycomb") architecture, smooth osseous expansion, and cortical remodeling, establishing its intraosseous origin and substantially narrowing the differential diagnosis. Histopathological examination confirmed cavernous hemangioma. This case highlights the complementary roles of CT and MRI in characterizing complex skull base lesions and demonstrates how recognition of characteristic osseous trabeculation, absence of aggressive bone destruction, and preservation of vascular patency despite encasement can differentiate PICH from jugular paraganglioma, schwannoma, meningioma, chordoma, chondrosarcoma, and metastatic disease. In conjunction with a review of the literature, this report expands the limited spectrum of jugular foramen PICH and underscores the importance of considering this rare entity in the differential diagnosis of destructive skull base lesions to facilitate accurate preoperative diagnosis and surgical planning.

## Introduction

Primary intraosseous cavernous hemangiomas (PICHs) are uncommon benign vascular tumors arising within bone and account for approximately 0.7-1% of all primary bone tumors and nearly 0.2% of skull tumors [1]. They predominantly involve the vertebral column and calvarium, whereas skull base involvement is distinctly uncommon. Within the skull base, reported lesions most frequently involve the sphenoid, petrous temporal bone, or clivus, with jugular foramen involvement being exceptionally rare. Their rarity, coupled with nonspecific clinical manifestations, often leads to delayed diagnosis and frequent misclassification as more common skull base neoplasms [1-4].

Patients typically present with slowly progressive symptoms related to compression of adjacent cranial nerves rather than rapid neurological deterioration. Hearing loss, pulsatile tinnitus, headache, lower cranial nerve dysfunction, vestibular symptoms, or cerebellopontine angle manifestations may occur depending on lesion location. Radiologically, these lesions demonstrate variable imaging appearances ranging from expansile osteolytic destruction to characteristic coarse trabecular or "honeycomb" architecture, with avid post-contrast enhancement reflecting their vascular nature. However, these classic imaging features are inconsistently observed in skull base lesions, making preoperative diagnosis particularly challenging. The characteristic coarse honeycomb/trabecular appearance of PICH on CT reflects remodeled residual bony trabeculae interspersed between dilated cavernous vascular channels; however, at the skull base, these classic trabeculations and well-defined expansile margins may be less conspicuous, contributing to diagnostic overlap with other hypervascular skull-base lesions. Consequently, jugular paraganglioma, schwannoma, meningioma, metastatic disease, chordoma, chondrosarcoma, eosinophilic granuloma, and other primary skull base tumors are frequently considered before intraosseous cavernous hemangioma [1-6].

We present a histopathologically confirmed PICH centered within the right jugular foramen with extensive petrous temporal bone involvement that radiologically simulated a jugular paraganglioma. The case highlights the complementary roles of high-resolution temporal bone computed tomography (HRCT), CT angiography, CT venography, and MRI in lesion characterization, demonstrates important imaging clues that may suggest the diagnosis preoperatively, and reviews the imaging-based differential diagnosis of destructive jugular foramen lesions along with a relevant review of the literature.

## Case presentation

A 46-year-old man presented to the otorhinolaryngology clinic with a five-year history of progressive right-sided hearing loss. Otoscopic examination revealed a normal external auditory canal and an intact tympanic membrane. Audiological evaluation demonstrated right-sided sensorineural hearing loss, suggesting a retrocochlear rather than middle ear pathology. Based on the clinical presentation, vestibular schwannoma, cerebellopontine angle meningioma, chronic labyrinthitis, and Ménière disease were initially considered, and magnetic resonance imaging (MRI) of the brain was performed for further evaluation.

MRI demonstrated a relatively well-defined lobulated lesion measuring 2.6 × 2.5 × 3.1 cm (AP × TR × CC) centered within the right cerebellopontine angle cistern, extending inferiorly into the right jugular foramen. The lesion appeared hypointense on T1-weighted images and heterogeneously hyperintense on T2/fluid-attenuated inversion recovery (FLAIR) sequences (Figures 1a-c).

**Figure 1:**
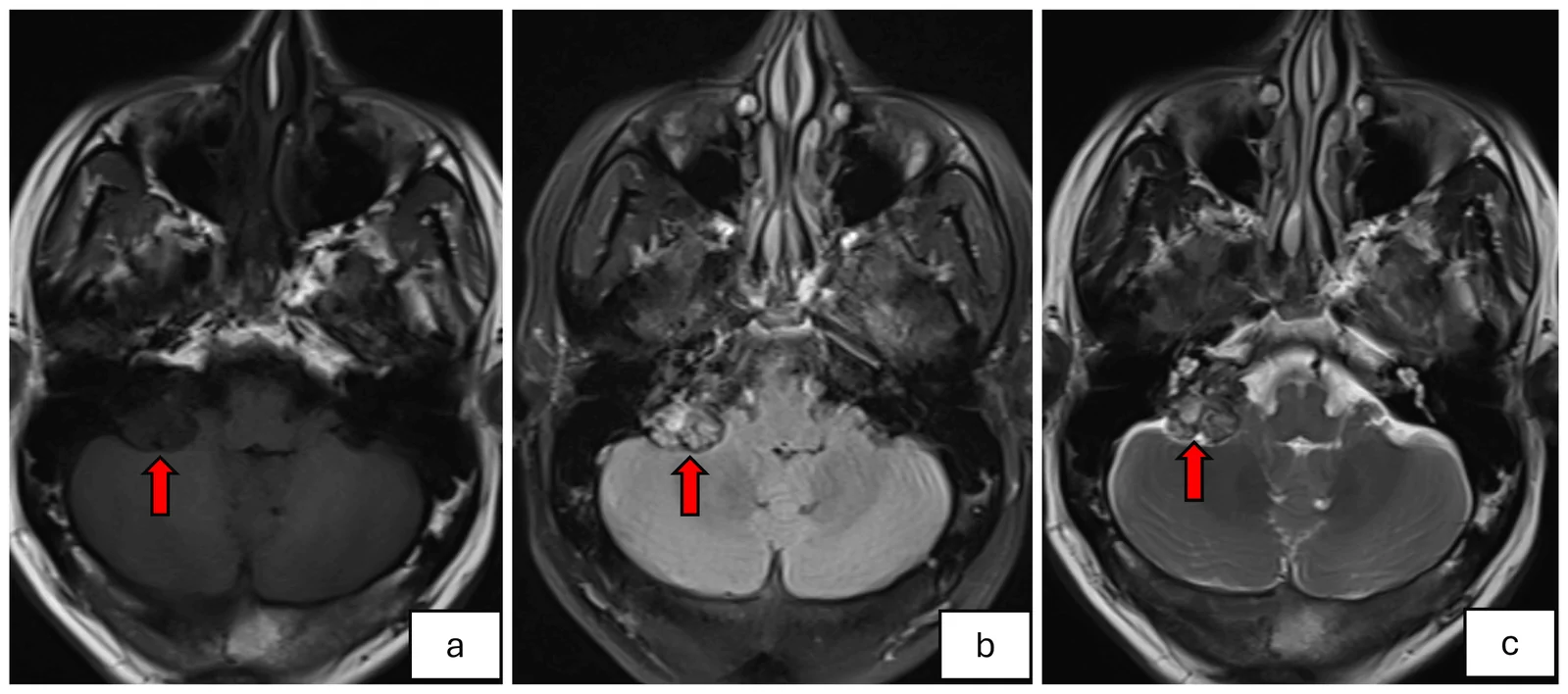
Magnetic resonance imaging of the brain demonstrating a relatively well-defined lobulated lesion centered in the right cerebellopontine angle cistern. a: Axial T1-weighted image shows the lesion (red arrow) to be hypointense, seen separately from the clivus. b: Axial FLAIR image (red arrow) reveals a heterogeneous hyperintense lesion abutting the cerebellum, with distinct margins from it. c: Axial T2-weighted image demonstrates a heterogeneous hyperintense lesion (red arrow) showing distinct planes from the brainstem, cerebellum, and inner ear.

Diffusion-weighted imaging (DWI) demonstrated no diffusion restriction, excluding a hypercellular lesion, while susceptibility-weighted imaging (SWI) revealed multiple blooming foci, suggestive of intralesional calcification or chronic blood products (Figures 2a-d).

**Figure 2:**
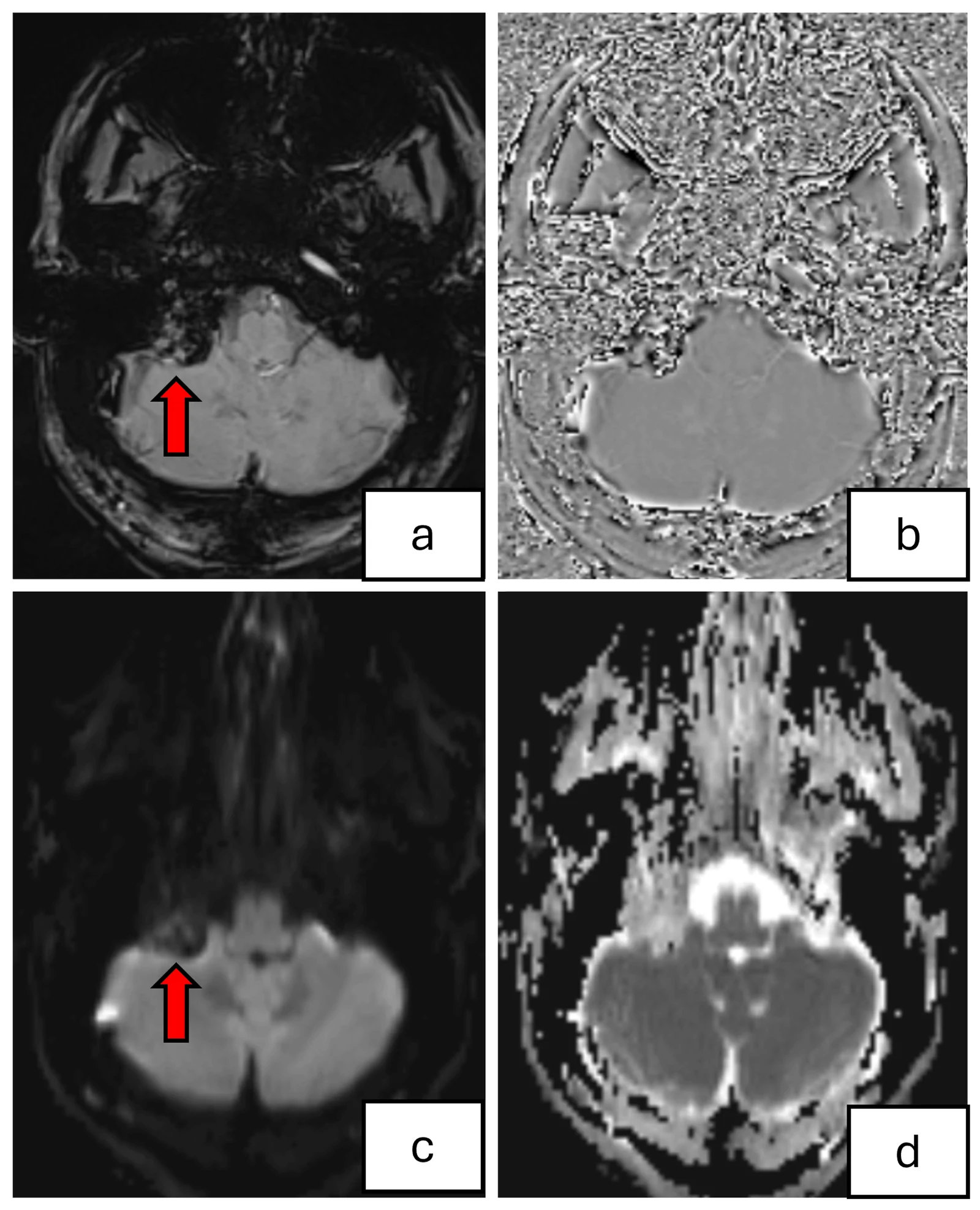
Susceptibility-weighted imaging (SWI) and diffusion-weighted imaging (DWI). a,b: Susceptibility-weighted imaging (SWI) reveals multiple intralesional blooming foci (red arrow) with corresponding mixed intensities on PHASE, suggestive of calcifications and/or blood products. c,d: Diffusion-weighted imaging (DWI) and corresponding ADC demonstrate no diffusion restriction (red arrow) within the lesion.

Evaluation of the cranial nerves in CISS (Constructive Interference in Steady State) sequence demonstrated preservation of the proximal cisternal segments of the facial and vestibulocochlear nerve complex, with anterior displacement of their distal portions (Figures 3a, 3b).

**Figure 3:**
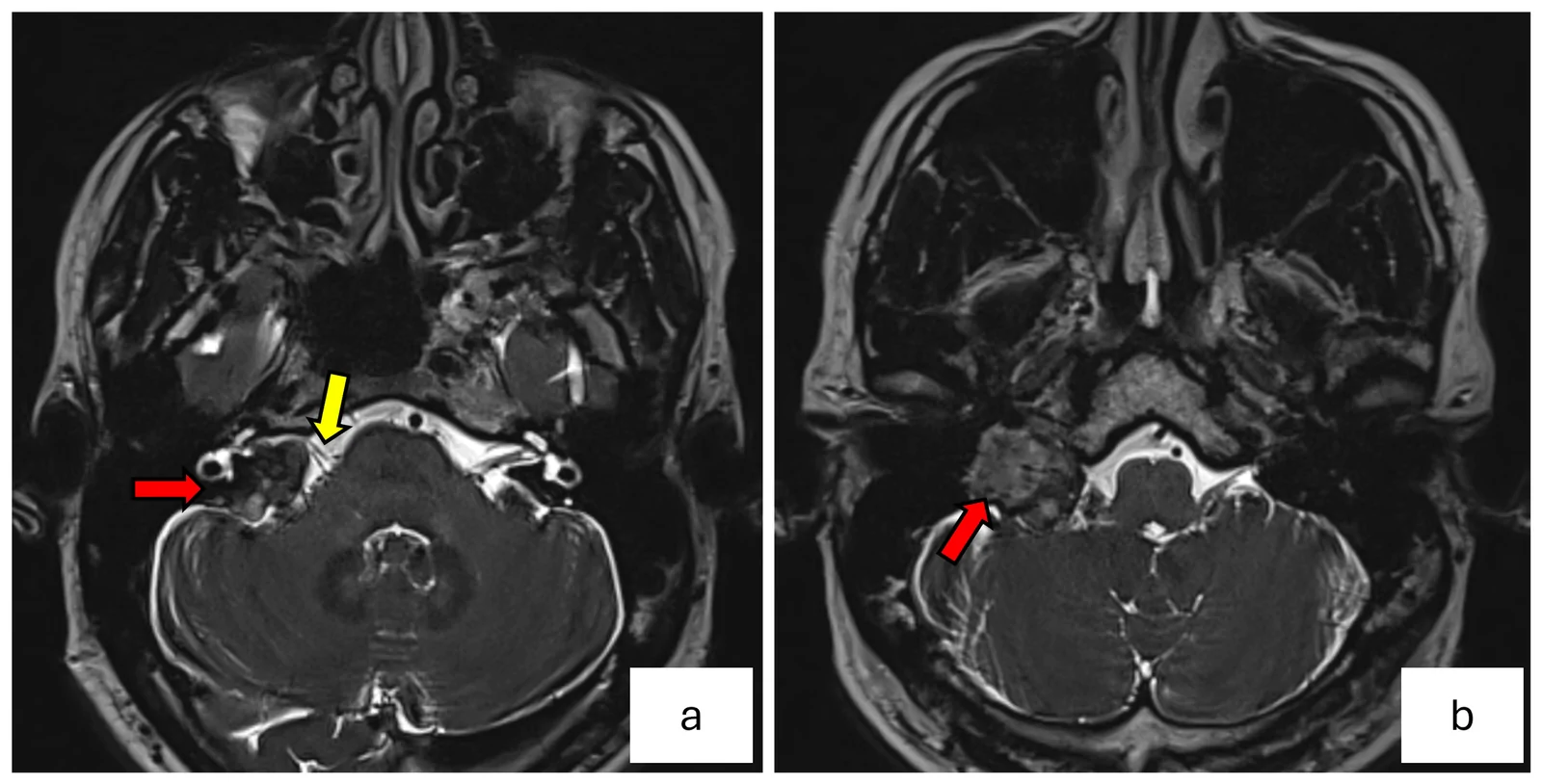
Constructive Interference in Steady State (CISS) MRI demonstrating the relationship of the lesion to the cranial nerves. a: The proximal cisternal segments of the right facial (VII) and vestibulocochlear (VIII) nerve complex are preserved (yellow arrow), with anterior displacement of cisternal segments by the lesion (red arrow). b: The proximal glossopharyngeal (IX), vagus (X), and accessory (XI) nerves are identified; however, their distal segments at the level of the right jugular foramen are obscured by the lesion (red arrow) on the right side.

The glossopharyngeal, vagus, and accessory nerves were visualized proximally; however, their distal segments at the level of the jugular foramen were obscured by the lesion. There was no involvement of the surrounding vascular structures. The lesion showed avid heterogenous enhancement on post-contrast images (Figures 4a-c).

**Figure 4:**
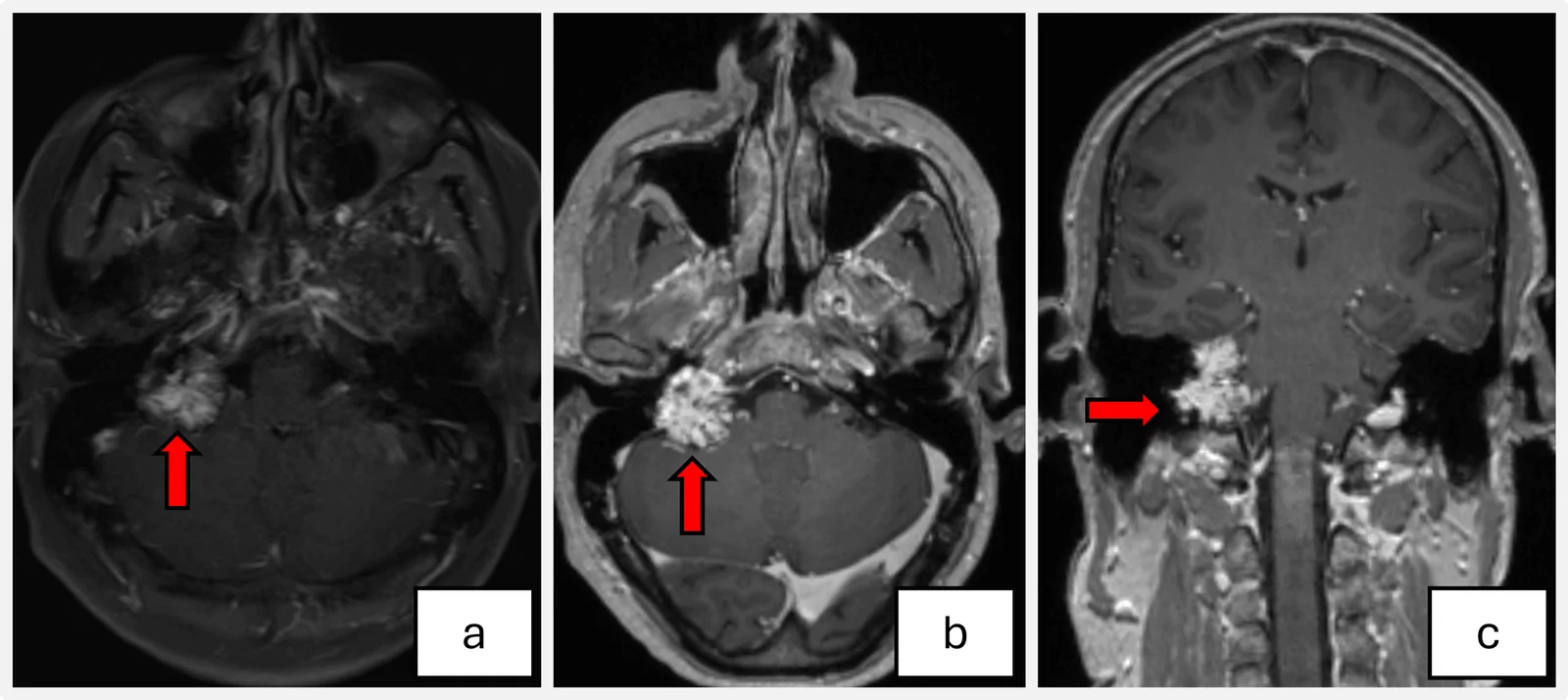
Post-contrast T1-weighted magnetic resonance images demonstrating avid heterogeneous enhancement of the lesion. a: Axial fat-saturated post-contrast T1-weighted image demonstrating feathery striations of the lesion (red arrow). b: Axial MPRAGE non-fat-saturated post-contrast T1-weighted image demonstrating avid heterogeneous enhancement of the lesion (red arrow). c: Coronal reconstructed non-fat-saturated post-contrast T1-weighted image, illustrating the inferior extension of the lesion into the right jugular fossa (red arrow).

Non-contrast computed tomography of the skull base demonstrated a well-defined expansile osseous lesion involving the jugular fossa, exhibiting a characteristic internal coarse trabecular ("honeycomb" or "soap-bubble") architecture with cortical thinning and smooth bony remodeling, without aggressive permeative destruction or a significant extraosseous soft-tissue component (Figures 5a-c). Contrast-enhanced computed tomography (CECT) demonstrated mild heterogeneous enhancement without identifiable enlarged feeding arteries (Figure 5d).

**Figure 5:**
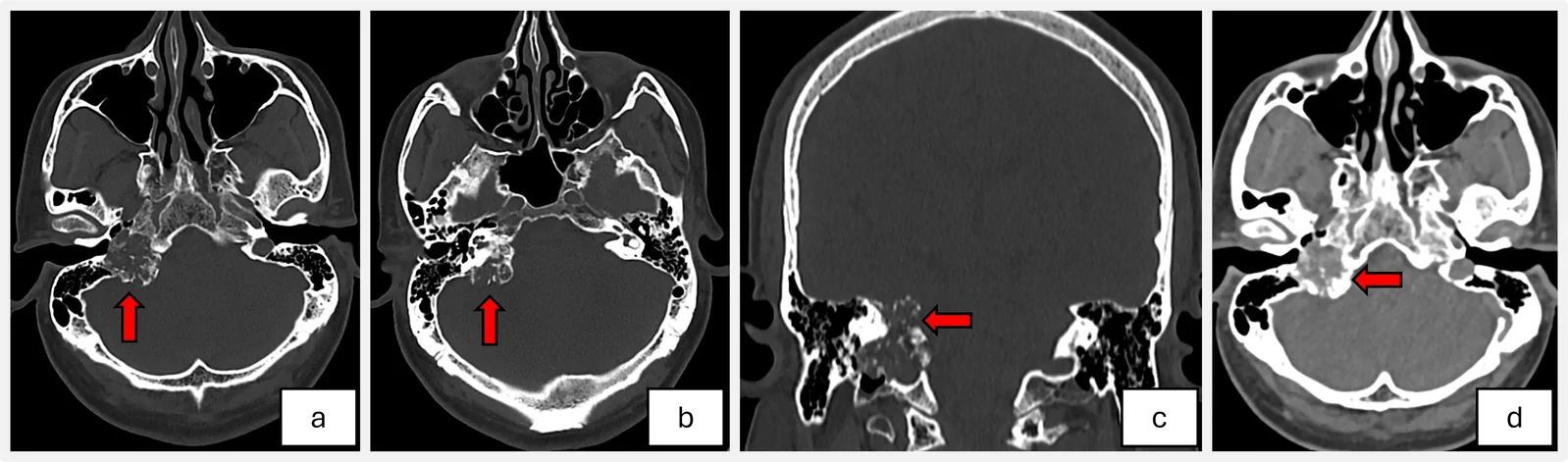
Skull base CT demonstrating an expansile osseous lesion centered in the skull base around the right petro-occipital region. a,b: Axial non-contrast CT (NCCT) shows characteristic coarse trabeculations with a "honeycomb"/"soap-bubble" appearance and smooth cortical remodeling. c: Coronal NCCT reconstruction demonstrates the extent of bony involvement from the petrous bone superiorly to the occipital condyles inferiorly. d: Axial contrast-enhanced CT (CECT) shows mild heterogeneous enhancement in the central soft-tissue component without enlarged feeding arteries.

Based on the multimodality imaging findings, the leading differential diagnoses included jugular paraganglioma, primary intraosseous meningioma, and low-grade chondrosarcoma, with a benign primary osseous lesion such as hemangioma also considered.

Following multidisciplinary discussion, the patient underwent right retro-mastoid craniotomy for tumor decompression. The lesion was predominantly heavily calcified and bony hard, interspersed with sparse but highly vascular soft-tissue components. The calcified portion was gradually drilled, and the vascular component was excised and submitted for histopathological examination. The operative findings correlated well with the preoperative imaging appearance. The patient had an uneventful immediate postoperative recovery and was discharged in stable condition.

Histopathological examination revealed mature bony trabeculae separated by dilated cavernous vascular spaces lined by a single layer of bland endothelial cells and filled with blood, consistent with an intraosseous cavernous hemangioma. Immunohistochemistry demonstrated diffuse CD34 positivity in the endothelial lining cells, confirming their vascular nature (Figures 6a, 6b).

**Figure 6:**
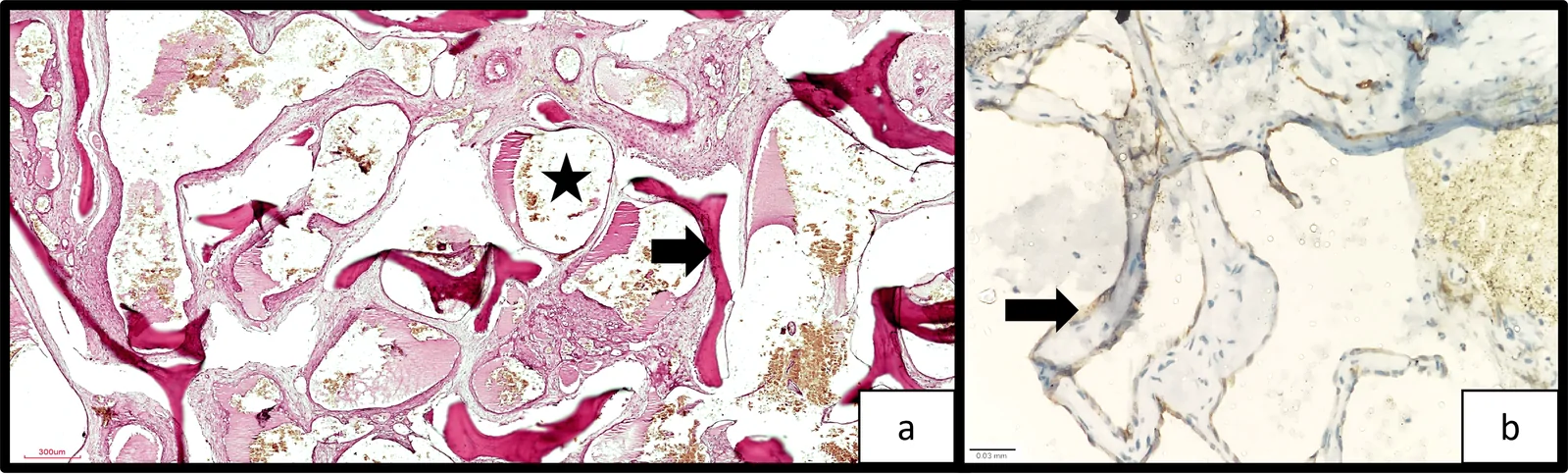
Histopathological images of the tissue specimen. a: The section examined shows bony trabeculae (black arrow) with intervening spaces composed of dilated cavernous spaces lined by benign endothelial cells and filled with blood (black star). b: Immunohistochemistry (IHC, 200×) for CD34 highlights the benign endothelial cells (black arrow).

## Discussion

PICH is a rare benign vascular neoplasm accounting for approximately 0.7-1% of all primary bone tumors and nearly 0.2% of skull tumors, with the vertebral column and calvarium representing the most frequent sites of involvement [1]. Skull base lesions are exceedingly uncommon, particularly those arising within the temporal bone, petrous apex, clivus, or jugular foramen. Consequently, most radiologists encounter these lesions only rarely, resulting in frequent preoperative misdiagnosis as more common skull base tumors [1,2].

Among reported skull lesions, the frontal and parietal bones account for the majority of cases, whereas temporal bone involvement constitutes only a small proportion. Even within the temporal bone, previously reported lesions have predominantly involved the internal auditory canal, geniculate ganglion, external auditory canal, or other part of the petrous bone rather than the jugular fossa [3-7]. The present case therefore represents an unusual anatomical location that further expands the limited spectrum of reported skull base intraosseous cavernous hemangiomas.

Clinical manifestations largely depend upon the anatomical site of involvement rather than the biological behavior of the lesion. Because these tumors enlarge slowly, symptoms generally develop gradually through progressive compression of adjacent cranial nerves or osseous structures. Headache, hearing loss, tinnitus, vestibular symptoms, and lower cranial nerve deficits have all been described in previous reports [1-3,8]. In the present case, the patient presented with a five-year history of progressive unilateral sensorineural hearing loss and headache without significant lower cranial nerve dysfunction, despite the lesion being centered around the jugular fossa region of the petrous bone. This relatively indolent presentation correlated well with the slow-growing benign nature of intraosseous cavernous hemangioma.

Radiologically, diagnosis of skull base PICH remains challenging because imaging characteristics vary according to the proportion of vascular channels, fibrous tissue, fatty marrow, calcification, and residual trabecular bone. Although the diagnosis is straightforward in classic calvarial lesions demonstrating a characteristic trabecular pattern, skull base lesions frequently lack these typical appearances because of complex surrounding anatomy and overlapping imaging features with other jugular foramen tumors [1-4].

Computed tomography remains the cornerstone for evaluating these lesions because it accurately demonstrates osseous remodeling and internal trabecular architecture. Classical intraosseous cavernous hemangiomas exhibit a well-defined expansile osteolytic lesion with preservation of cortical margins and coarse thickened trabeculae producing the characteristic "honeycomb," "soap-bubble," or "sunburst" appearance [1,2,5,7]. These trabeculae correspond histologically to remodeled residual bony struts interspersed between dilated vascular channels. Unlike aggressive malignant lesions, cortical thinning usually predominates over frank permeative destruction, and periosteal reaction is typically absent. Our case demonstrated several of these characteristic CT findings [1,2].

These imaging findings closely resemble those described by Liu et al., who reported a sphenoid PICH demonstrating the classic trabeculated honeycomb appearance on CT, and by Tashiro et al., who described coarse bony trabeculation within a clival lesion. Recognition of this internal trabecular architecture proved to be the single most important imaging clue suggesting an intraosseous vascular lesion rather than an aggressive skull base neoplasm [2,7].

MRI findings are considerably less specific but provide important information regarding lesion extent, cranial nerve relationships, vascular encasement, and intracranial extension. Most reported skull base PICHs demonstrate iso- to hypointense signal on T1-weighted imaging, heterogeneous hyperintensity on T2-weighted sequences, and avid but heterogeneous post-contrast enhancement. Variable T1 hyperintensity may occur secondary to intralesional fat or subacute hemorrhage, whereas marked T2 hyperintensity reflects slow-flowing blood within dilated cavernous vascular spaces [1,2,4-6].

The MRI appearance in our patient closely paralleled previously reported skull base lesions. The mass demonstrated T1 hypointensity, heterogeneous T2 hyperintensity, and heterogeneous enhancement following contrast administration. DWI showed no diffusion restriction, excluding hypercellular tumors such as lymphoma, while SWI demonstrated multiple blooming foci corresponding to calcification or chronic hemorrhagic products. These susceptibility findings have received relatively little emphasis in previous reports but provided an additional clue supporting a chronic vascular lesion rather than schwannoma or meningioma.

Another notable feature was preservation of the proximal VII-VIII nerve complex with displacement rather than infiltration of adjacent cranial nerves or vascular invasion, favoring an osseous epicenter over a primary cranial nerve tumor. Previous reports have likewise shown that PICHs remodel and displace adjacent neurovascular structures while preserving vascular patency, reflecting their benign biological behavior [2-4].

The lesion initially suggested a jugular paraganglioma on MRI, because of its location and enhancement pattern. However, unlike paragangliomas, which typically show intense homogeneous enhancement, multiple flow voids producing the classic "salt-and-pepper" appearance, enlarged feeding arteries, and permeative destruction of the jugular foramen, our lesion did not demonstrate any of these features.

Lower cranial nerve schwannoma was another important differential because of the jugular foramen extension. However, schwannomas characteristically present as soft-tissue masses causing neural foraminal widening rather than expansile osseous remodeling with coarse trabeculation. Preservation of the proximal cranial nerves with displacement of the distal segments further argued against a nerve sheath neoplasm [3-6].

Primary intraosseous meningioma, low-grade chondrosarcoma, chordoma, hypervascular metastasis, and fibrous dysplasia were also considered. Unlike intraosseous meningiomas, which are dural-based and typically demonstrate hyperostosis and homogeneous enhancement, our lesion was expansile, heterogeneously enhancing, and lacked dural involvement [1-3,6,8]. Chondrosarcoma and chordoma usually exhibit aggressive bone destruction, an exuberant soft-tissue component, and a characteristic chondroid matrix or clival origin, while hypervascular metastases show destructive margins, marrow replacement, and extraosseous extension in patients with known malignancy. Fibrous dysplasia typically demonstrates diffuse bone expansion with a homogeneous ground-glass matrix and minimal enhancement rather than the coarse honeycomb trabeculation as seen in our case [1-5,7].

A key observation was the discrepancy between MRI and CT findings. MRI suggested a CP angle soft-tissue lesion with extension into the jugular foramen because of the lesion's location and enhancement, whereas HRCT established its intraosseous origin by demonstrating preserved cortical margins and characteristic coarse trabecular architecture. This highlights the complementary roles of MRI and CT in skull base lesions [1-3].

Operative and histopathological findings closely correlated with imaging, demonstrating remodeled bony trabeculae interspersed with cavernous vascular channels, explaining the characteristic CT and MRI appearance. This excellent radiologic-pathologic concordance underscores the diagnostic value of multimodality imaging in these rare lesions.

Previous reports have consistently emphasized the rarity of skull base intraosseous cavernous hemangiomas. Liu et al. described a sphenoid skull base lesion in which CT demonstrated the characteristic trabeculated appearance, while MRI showed heterogeneous enhancement similar to our case [2]. Tashiro et al. reported a clival lesion, highlighting the importance of CT in identifying the internal osseous architecture and differentiating the lesion from chordoma [7]. Sade et al. described petrous temporal bone involvement presenting with hearing-related symptoms and cranial nerve compression, closely resembling the clinical presentation in our patient [3]. More recently, Torii et al. reported a temporal bone intraosseous hemangioma presenting as progressive hearing impairment, emphasizing that these lesions should be considered whenever an expansile temporal bone lesion demonstrates characteristic trabecular remodeling on CT [4]. Reports by Kanaan et al. and Branco et al. further illustrate that extra-axial and skull base cavernous hemangiomas may closely simulate meningioma or other skull base tumors on MRI, with definitive diagnosis ultimately established by histopathology [5,8]. Collectively, these reports and our case demonstrate that despite variable MRI appearances, careful assessment of CT morphology frequently provides the key to an accurate preoperative diagnosis.

To our knowledge, involvement of the petrous bone around the jugular fossa by a PICH remains exceptionally uncommon. Most previously reported lesions have arisen from the frontal, parietal, sphenoid, or clival bone, whereas a lesion centered within the petrous part of the jugular fossa has been only rarely documented. The present case therefore expands the imaging spectrum of skull base intraosseous cavernous hemangiomas.

Table 1 succinctly summarizes the principal imaging features distinguishing PICH from other jugular foramen lesions and highlights the findings that favored the diagnosis in the present case [1-8].

**Table 1:** Imaging Differentials of Jugular Foramen Lesions PICH: primary intraosseous cavernous hemangioma; CT: computed tomography; CN: cranial nerve; MRI: magnetic resonance imaging; SWI: susceptibility-weighted imaging; DWI: diffusion-weighted imaging

Feature	Present Case (PICH)	Jugular Paraganglioma	Lower CN Schwannoma	Primary Intraosseous Meningioma	Chondrosarcoma	Chordoma	Hypervascular Metastasis
Age	Middle-aged	40–70 yrs	30–60 yrs	Middle-aged	30–60 yrs	40–70 yrs	Variable
Epicenter	Bone (jugular foramen)	Jugular bulb	Cranial nerve	Bone/dura	Petro-occipital synchondrosis	Clivus	Bone
CT	Honeycomb/soap-bubble trabeculae	Permeative erosion	Smooth foraminal widening	Hyperostosis/lysis	Ring-and-arc calcification	Lytic destructive	Aggressive lysis
Bone remodeling	Smooth expansile	Destructive	Mild expansion	Hyperostotic	Destructive	Marked destruction	Destructive
MRI T2	Heterogeneous hyperintense	Markedly hyperintense	Hyperintense	Iso-hypointense	Very hyperintense	Very hyperintense	Variable
Enhancement	Moderate heterogeneous	Intense avid	Moderate	Homogeneous	Septal/peripheral	Heterogeneous	Variable
Flow voids	Absent	Present (salt-pepper)	Absent	Absent	Absent	Absent	Variable
SWI	Blooming (calcification/hemorrhage)	Flow-related signal voids	Rare	Calcification occasionally	Calcification	Variable	Hemorrhage possible
DWI	No restriction	No restriction	No restriction	Usually none	Usually none	Mild restriction possible	Variable
Key feature	Trabecular honeycomb bone architecture	Hypervascular lesion with feeding vessels	Nerve origin	Dural attachment/hyperostosis	Chondroid matrix	Midline clival origin	Known primary malignancy

## Conclusions

PICH of the jugular fossa in the petrous bone is an exceptionally rare benign lesion that can closely mimic jugular paraganglioma and other skull base tumors. Recognition of characteristic CT findings, particularly coarse honeycomb trabecular architecture with smooth expansile osseous remodeling, in conjunction with MRI assessment of cranial nerve and vascular relationships, is critical for accurate preoperative diagnosis. SWI may provide an additional diagnostic clue by demonstrating intralesional blooming foci and may serve as a useful adjunct in the evaluation of such lesions. Accurate preoperative recognition of PICH can distinguish it from hypervascular mimics such as paraganglioma, thereby influencing surgical planning and potentially avoiding unnecessary preoperative interventions such as embolization. This case highlights the importance of multimodality imaging and radiologic-pathologic correlation in narrowing the differential diagnosis and guiding appropriate management. However, given the recent nature of this single case and limited long-term follow-up, assessment of long-term surgical outcomes remains limited.
